# Proteomic signature of tubulointerstitial tissue predicts prognosis in IgAN

**DOI:** 10.1186/s12882-022-02736-4

**Published:** 2022-03-24

**Authors:** Flavia Teodora Ioana Paunas, Kenneth Finne, Sabine Leh, Hans-Peter Marti, Frode Berven, Bjørn Egil Vikse

**Affiliations:** 1 Department of Medicine, Haugesund Hospital, Haugesund, Norway; 2 Department of Clinical Medicine, University of Bergen, Bergen, Norway; 3 Department of Pathology, Haukeland University Hospital, Bergen, Norway; 4 Department of Medicine, Haukeland University Hospital, Bergen, Norway; 5 Department of Biomedicine, University of Bergen, Bergen, Norway

**Keywords:** IgA nephropathy, ESRD, Formalin-fixed paraffin embedded kidney biopsy tissue, Liquid chromatography–tandem mass spectrometry, Proteomic analyses, Tubulointerstitium

## Abstract

**Background:**

IgA nephropathy (IgAN) is associated with a significant risk of progression to kidney failure. Tubular atrophy is an established important risk factor for progressive disease, but few studies have investigated tubulointerstitial molecular markers and mechanisms of progression in IgAN.

**Methods:**

Based on data from the Norwegian Renal Registry, two groups were included: IgAN patients with (*n* = 9) or without (*n* = 18) progression to kidney failure during 10 years of follow-up. Tubulointerstitial tissue without discernible interstitial expansion or pronounced tubular alterations was microdissected, proteome was analysed using tandem mass spectrometry and relative protein abundances were compared between groups.

**Results:**

Proteome analyses quantified 2562 proteins with at least 2 unique peptides. Of these, 150 proteins had significantly different abundance between progressive and non-progressive IgAN patients, 67 were more abundant and 83 less abundant. Periostin was the protein with the highest fold change between progressive and non-progressive IgAN (fold change 8.75, *p* < 0.05) and periostin staining was also stronger in patients with progressive vs non-progressive IgAN. Reactome pathway analyses showed that proteins related to inflammation were more abundant and proteins involved in mitochondrial translation were significantly less abundant in progressive vs non-progressive patients.

**Conclusions:**

Microdissection of tubulointerstitial tissue with only mild damage allowed for identification of proteome markers of early progressive IgAN. Periostin abundance showed promise as a novel and important risk marker of progression.

**Supplementary Information:**

The online version contains supplementary material available at 10.1186/s12882-022-02736-4.

## Background

The clinical course of IgA nephropathy (IgAN) is variable, ranging from asymptomatic to rapid progression to kidney failure. Tubular atrophy is one of the most powerful prognostic factors in IgAN and is included in the MEST score of the Oxford Classification [1].

The mechanisms of tubular damage in IgAN is not fully understood, but it has been suggested that proteinuria, monocyte/macrophage infiltration, and glomerulotubular cross-talk are important players [2]. In addition, tubular epithelial cells (TEC) actively participate by synthesizing inflammatory mediators (cytokines and chemokines) and reactive oxygen species (ROS) [3]. The cytokines and chemokines can further recruit inflammatory cells in the interstitium which will also secrete pro-inflammatory, pro-fibrotic factors and matrix metalloproteinases contributing to inflammation and fibrosis [4].

It is accepted that the kidney tubuli play a central role in the progression of chronic kidney disease [5], but most of our knowledge of molecular mechanisms of chronic tubular damage stems from animal models. It is generally believed that mechanisms of tubulointerstitial damage are similar across species and human renal diseases but differences could exist. From a morphological perspective, the tubulointerstitial alterations in progressive IgAN are similar to those found in other chronic kidney diseases, again suggesting that underlying mechanisms might be similar [6], but a better understanding of the underlying mechanisms that lead to these alterations are needed.

Understanding the molecular changes that occur in the tubulointerstitium of patients with IgAN is an important step to understanding the progression of IgAN to kidney failure. In the present study we investigated the proteomic changes of microdissected tubulointerstitial tissue with only mild-moderate damage from patients with progressive vs non progressive IgAN.

## Methods

### Registry data used in the study

Data from the Norwegian Kidney Biopsy Registry were used for patient selection and these data were available from 1988. Data on kidney failure were retrieved from the Norwegian Renal Registry and these data were available from 1980 until 2013. Formally, these two registries merged in 2016 to the Norwegian Renal Registry.

Serum creatinine was measured using kinetic Jaffe method before 2005 and for use in the present study we recalculated it based on a formula used by Hallan et al. to IDMS-traceable values [7]. We calculated estimated glomerular filtration rate (eGFR) based on the CKD-EPI equation [8]. Urinary protein was quantified as grams/24 h either from directly measured values, by calculation from reported urinary protein to creatinine ratio or if only reported by urinary dipstick a negative dipstick was set to 0 g/24 h, 1 + was set to 0.5 g/24 h, 2 + was set to 1.0 g/24 h and 3 + was set to 3.0 g/24 h.

### Study population

The study was approved by the Regional Committee for Medical and Health Research Ethics (approval number 2013/553).. In total 27 IgAN patients were included in the study, these were divided based on whether or not they progressed to kidney failure. The inclusion criteria were: (1) for Non-progressive IgAN: diagnosis of IgAN at kidney biopsy, eGFR > 45 ml/min/1.73 m2, urinary protein > 0,5 g/24 h and no development of ESRD during a follow-up period of at least 10 years. (2) for Progressive IgAN: diagnosis of IgAN at kidney biopsy, eGFR > 45 ml/min/1.73 m2, urinary protein < 2 g/ 24 h and development of ESRD during the first 10 years after kidney biopsy. Of the 27 patients, 18 did not progress to kidney failure during the first 10 years after diagnosis and 9 progressed to kidney failure. For the present study we included the IgAN patients who had available kidney biopsy tissue that allowed for microdissection of tubulointerstitial tissue with only mild changes (described in detail below).

None of the patients had known preexisting hypertension. The main indications for biopsy in the IgAN group were hematuria, proteinuria or reduced GFR.

### Laser capture microdissection and sample preparation

**Fig. 1:**
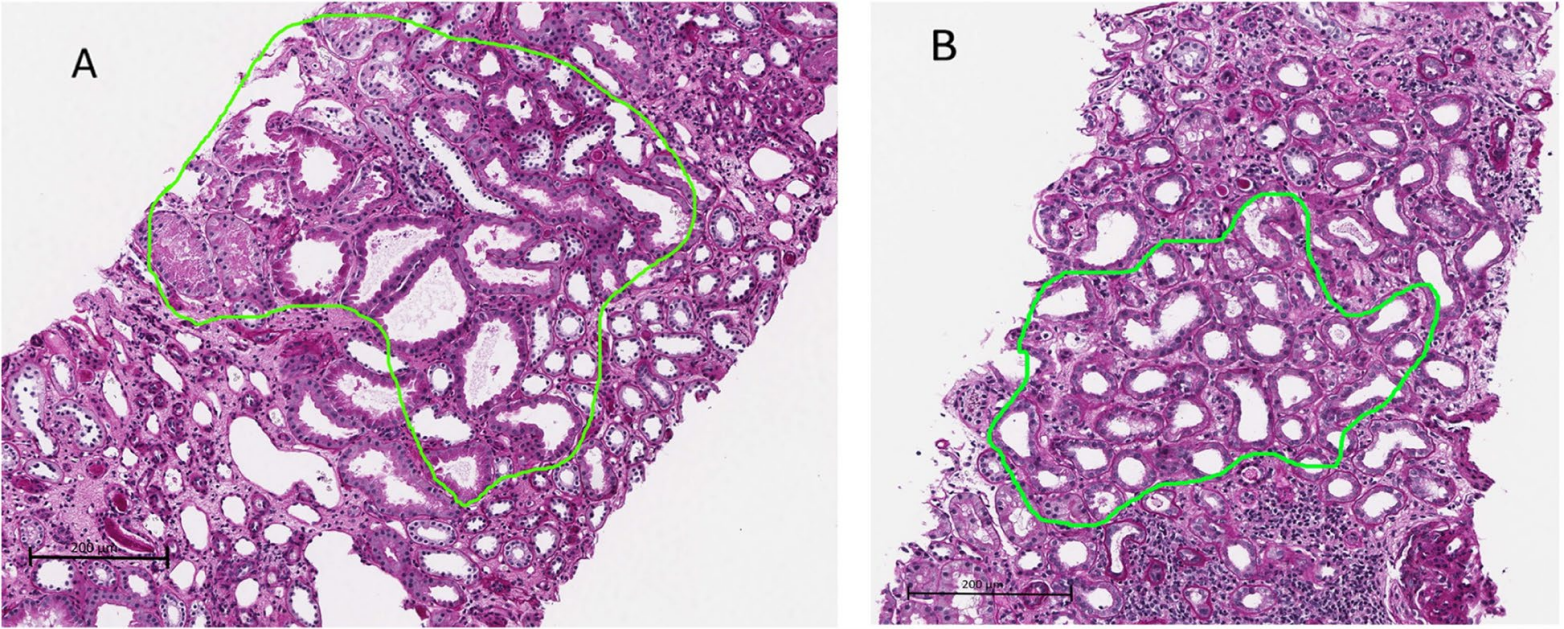
PAS staining in the two patients group. Area of tubulointerstitium eligible for microdissection (selected with green) (**A**-non-progressive IgAN and **B**-progressive IgAN)

Sample preparation was done as previously described [9]. In short, formalin-fixed paraffin-embedded (FFPE) tissue that was left after the diagnostic biopsy examination was cut into five micrometer thick sections, and approximately 3 million square micrometer tubulointerstitial tissue were laser microdissected and collected into one tube per biopsy. Tubulointerstitial areas with tubular atrophy or areas with interstitial expansion were not microdissected (Fig. 1). Although parts of the kidney biopsies consist of such areas, other parts does not have these characteristics. Sample handling and protein extraction were done as previously described [9].

### Liquid chromatography and tandem mass spectrometry and data analysis

Q-Exactive HF (Thermo Scientific) connected to a Dionex Ultimate NCR-3500RS liquid chromatography (LC) system was used to analyze the samples, as previously described [9].

### Statistics

The relative differences in protein abundance between the progressive vs non-progressive groups are given as fold change. Statistical analysis of proteins abundance differences between groups was performed with Student’s t-test on log transformed intensity data. For other analyses, mean ± standard deviation (SD) is given. Standard two-sided t-tests were used, and *p*-values < 0.05 were considered statistically significant. A combined abundance score of the 7 proteins with highest fold change were calculated by multiplying the logarithmically transformed abundances for these 7 proteins. This score was tested as a possible classifier using receiver operating characteristic (ROC) statistics together with other potential classifiers such as individual protein abundance, other combinations of protein abundances and 1/eGFR.

### Pathway analysis

Pathway analysis were performed using the free web version of Reactome biological pathway analysis (https://reactome.org/). We tested which pathways that were upregulated in patients with progressive disease by including proteins that had significantly higher abundance in progressive vs non-progressive IgAN, and opposite for pathways that were downregulated, We used a *p* value < 0.05 for statistical significance.

Gene sets significantly enriched between progressive and non-progressive IgAN were explored by Gene Set Enrichment Analysis (GSEA) [10] applying the C5 gene onthology sets from the Molecular Signatures Database (MSigDB).

### Histology and immunohistochemistry

As in our previous study [9], the biopsies were scored in a blinded manner by an experienced nephropathologist (SL) using the Oxford classification scoring system. Oxford Score was performed retrospectively after case selection. Immunohistochemistry was performed on 3 µm thick sections from FFPE tissue after antigen retrieval with proteinase digestion. Immunohistochemistry for periostin (Sigma HPA012306, 1:50) was performed after heat-induced antigen retrieval. The tissue glass slides were scanned with ScanScope® XT (Aperio) at × 40. The slides were viewed in ImageScope 12. Periostin expression was quantified by image analysis with Positive pixel Count V9 (Aperio/Leica). Three tubulointerstitial square regions (total areal average 209290 square micrometer) were randomly chosen from each biopsy and analyzed for pixel intensity. The sum of average number of positive and average number of strong positive was divided by the area and compared between groups.

## Results

### Patient characteristics

The clinical and morphological characteristics of the patient groups at time of biopsy are summarized in Table 1. We found no differences in the clinical characteristics, but patients with progressive IgAN had a significantly higher T score than patients with non-progressive IgAN (all patients with non-progressive IgAN had T score 0). For progressive IgAN patients,, one patient had T score of 2 and three patients had T score of 1. The patient with a T-score of 2 showed a rapid progression to ESRD only two years after diagnosis. The three patients with a T-score of 1 progressed to ESRD three and half, six and seven years after diagnosis.

**Table 1:** Patient characteristics at time of biopsy

	IgAN without progression	IgAN with progression
*N*	18	9
Year of biopsy	1996.2 ± 3.3	1998.3 ± 5.8
Proportion male	83.3%	55.6%
Age (years)	32.6 ± 14.7	28.2 ± 10.8
Serum creatinine at time of biopsy (mmol/l)	83.8 ± 21.8	97.6 ± 21.4
Estimated glomerular filtration rate ª (ml/min/1.73 m^2^)	107.8 ± 27	88.4 ± 31.8
Systolic blood pressure (mmHg)	126.9 ± 13.6	132.1 ± 26.6
Diastolic blood pressure (mmHg)	78.4 ± 11.1	81.0 ± 12.1
Urinary protein (grams/24 h)	1.7 ± 1	1.5 ± 0.9
Body mass index (kg/m^2^)	24.0 ± 1.7	22.5 ± 4.2
No of years of follow-up	16.7 ± 3.4	
No of years from biopsy to kidney failure		5.2 ± 1.8
Percentage with M-score of 1^b^	35.3%	44.4%
Percentage with E-score of 1^b^	35.3%	22.2%
Percentage with S-score of 1^b^	52.9%	55.6%
Percentage with T-score of 1 or 2^b^	0%	44.4% *

*Significantly changed as compared to non progressive IgAN (*p* < 0.05)

ªEstimated by CKD EPI equation

^b^MEST score in Oxford classification

High-dose steroid treatment during the follow-up period was registered for 16 non-progressive IgAN patients and 6 progressive patients. One patient from each group received high-dose steroid treatment with at least 20 mg prednisolone daily for at least 1 month. Patients with progressive IgAN progressed to kidney failure, on average, 5.2 ± 1.8 years after diagnosis/biopsy, with the fastest progressor only after 2 years from time of biopsy and slowest after 8 years.

None of the patients had known hypertension, diabetes, hepatitis or vasculitis (data not shown).

### Proteomic analysis

From the quantitative proteomic analyses we identified a total of 2564 proteins with two or more unique peptides. Of these, 150 proteins were differentially abundant between progressive and non-progressive IgAN (p < 0.05);, 67 were more abundant and 83 less abundant in the progressive IgAN group. The proteins with highest fold change in progressive versus non-progressive IgAN were periostin (fc 8.75, *p* 0.0002), cathepsin G (fc 7.50, p 0.002) and filamin C (fc 3.42, p 0.03) (Table 2).

**Table 2:** List of the proteins with fold change above 1.5 between progressive and non-progressive IgAN. Only significantly changed proteins in list

Protein IDs	Gene name	*N* unique peptides	Protein names	Fold change	*P*-value
Q15063	POSTN	26	Periostin	8.75	0.0002
P08311	CTSG	5	Cathepsin G	7.50	0.002
Q14315	FLNC	17	Filamin-C	3.42	0.03
O14558	HSPB6	6	Heat shock protein beta-6	2.55	0.01
O95486	SEC24A	7	Protein transport protein Sec24A	2.40	0.02
P15088	CPA3	5	Mast cell carboxypeptidase A	2.21	0.03
P13796	LCP1	15	Plastin-2	2.12	0.04
Q71DI3	HIST2H3A	15	Histone H3.2	1.91	0.03
P07686	HEXB	20	Beta-hexosaminidase	1.89	0.03
P01023	A2M	33	Alpha-2-macroglobulin	1.82	0.03
P0CG05	IGLC2	10	Ig lambda-2 chain C regions	1.78	0.01
P39059	COL15A1	12	Collagen alpha-1(XV) chain;	1.74	0.003
P43251	BTD	2	Biotinidase	1.67	0.03
P80188	LCN2	6	Neutrophil gelatinase-associated lipocalin	1.67	0.00
Q6UX53	METTL7B	10	Methyltransferase-like protein 7B	1.61	0.03
P55899	FCGRT	2	IgG receptor FcRn large subunit p51	1.57	0.005
Q96S96	PEBP4	7	Phosphatidylethanolamine-binding protein 4	1.56	0.02
P20774	OGN	9	Mimecan	1.52	0.03
Q92820	GGH	11	Gamma-glutamyl hydrolase	1.51	0.05
P09668	GTSH	10	Cathepsin H	1.50	0.02

**Fig. 2:**
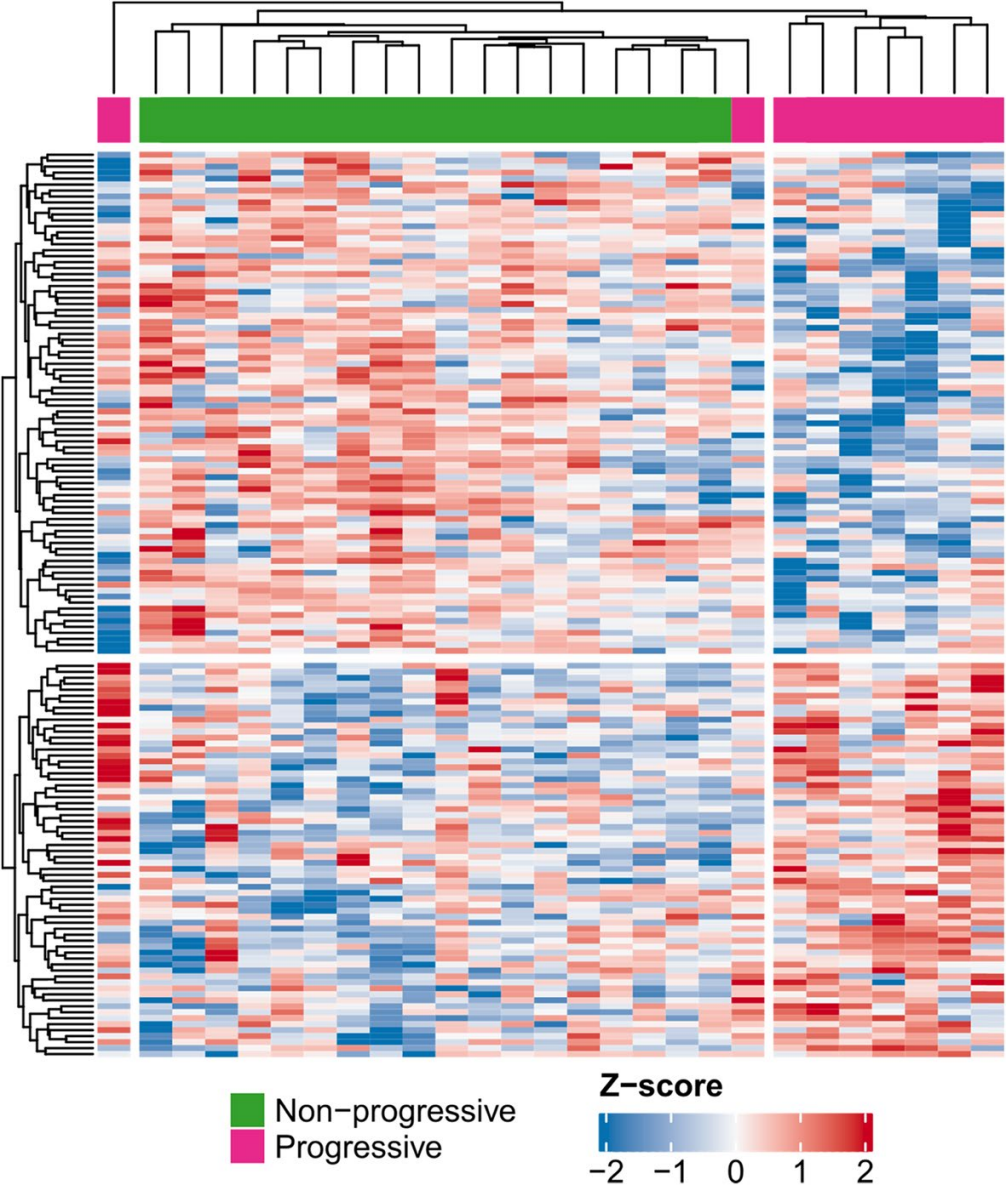
Unsupervised hierarchical clustering for proteins with significantly different abundance in progressive vs non-progressive IgAN. Each vertical bar represents a patient (purple progressive and green non-progressive) and each horizontal bar represents a protein (red indicates more abundant proteins while blue less abundant)

Unsupervised hierarchical clustering of the 150 proteins with significantly different abundance between patients with progressive and non-progressive IgAN showed a clear separation between progressive and non-progressive patients were seen, but there was some overlap (Fig. 2).

We further compared the proteomic data from tubuli with those from glomeruli [11] and found 4 common proteins which were more abundant in both compartments between patients with progressive vs non-progressive IgAN: periostin, filamin-C, platelet-activating factor acetylhydrolase IB subunit alpha and Ig alpha-1 chain C region. Three proteins were less abundant between progressive vs non-progressive IgAN in both compartments: AP-2 complex subunit mu, mitochondrial ATP synthase subunit alpha, and mitochondrial Enoyl-CoA hydratase domain-containing protein 2.

### Biological pathways

To investigate the systemic biological changes between progressive and non-progressive IgAN, we performed a pathway analysis using the Reactome pathway analysis tool (reactome.org). Pathways that were up-regulated in progressors were the innate immune system (18 significantly different proteins) and signaling by Rho GTPases (10 proteins) (Table 3). Pathways that were down-reulated in progressors were translation of proteins (18 proteins) and mitochondrial translation (10 proteins). These data were further supported by gene set enrichment analysis (GSEA) demonstrating that biological pathways related to mitochondrial translation, immune response and protein synthesis were the enriched pathways (Supplemental data). We compared these results with data from microdissected glomeruli from the same patient cohort and found that pathways associated with the immune system was also increased in glomeruli of patients with progressive IgAN as compared with non-progressive (19 proteins).

**Table 3:** Reactome pathways significantly associated with progression of IgAN. Up-regulated pathways tested by including proteins with significantly higher abundance in progressive vs non-progressive IgAN, and down-regulated pathways by including proteins with significantly lower abundance

Pathway name	N proteins changed abundance	*P*-value	FDR adjusted *p*-value
**Up-regulated pathways**			
Immune System	23	0.001	0.04
Innate Immune System	18	0.0001	0.01
Neutrophil degranulation	12	0.00002	0.01
Signaling by Interleukins	10	0.00002	0.03
Signaling by Rho GTPases	10	0.0002	0.02
RHO GTPase Effectors	9	0.00006	0.01
Vesicle-mediated transport	12	0.001	0.04
**Down-regulated pathways**			
Metabolism of proteins	23	0.02	0.3
Translation	18	4.5E-12	1.78E-09
Mitochondrial translation	10	2.2E-9	2.95E-07
tRNA Aminoacylation	5	0.00002	0.001
Mitochondrial tRNA aminoacylation	3	0.0005	0.023
Protein localization	6	0.001	0.053
Mitochondrial protein import	3	0.01	0.2

By comparing our dataset to the dataset of Epithelial-mesenchymal-transition (EMT) proteins found online: dbEMT 2.0 downloaded 19.01.21 (http://dbemt.bioinfo-minzhao.org/download.cgi) we found a total of 14 EMT proteins significantly different between progressive and non progressive IgAN, 11 proteins were more abundant and 3 less.

### Periostin staining

**Fig. 3:**
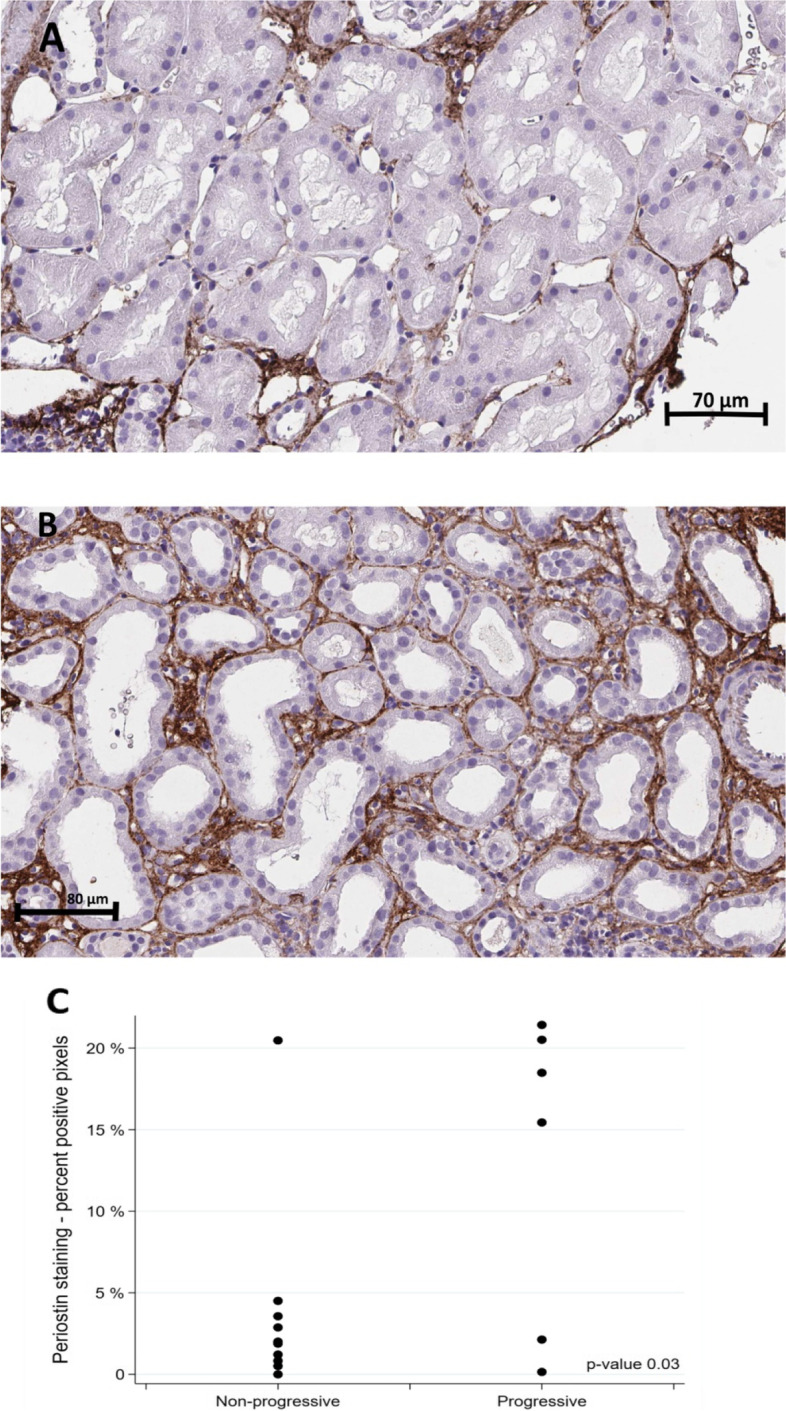
Periostin staining in the two patient groups (**A**) Non-progressive IgAN patient (**B**) Progressive IgAN patient and (**C**) Scatter plot illustrating Periostin staining between groups

Immunohistochemistry (IHC) for periostin was performed for 11 non-progressive and 6 progressive IgAN patients. As shown in Fig. 3, the staining was mostly peritubular, although there were also differences in tubular staining, and patients with progressive disease had more positivity than patients with non-progressive disease. This was supported by comparing the proportion of positive pixels between groups (mean proportion of positive pixels IgAN with progression was 13.0 vs 3.8 for patients without progression, *p* = 0.03).

### Classifier of progressive versus non-progressive IgAN

**Fig. 4:**
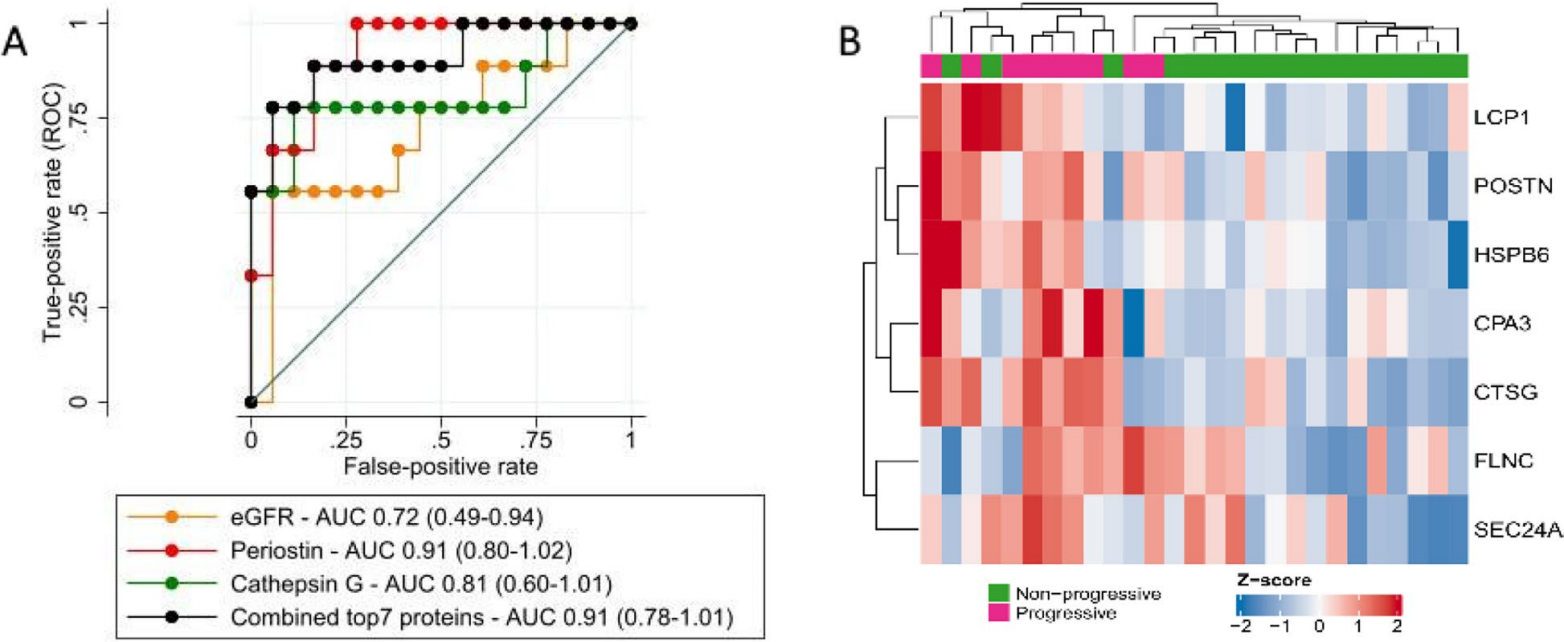
**A** Receiver Operating characteristic (ROC) plots for Periostin, Cathepsin G, combined top7 proteins (7 top proteins of Table 2) and eGFR. **B** Unsupervised hierarchical clustering for the 7 proteins with highest fold change difference in abundance between progressive vs non-progressive IgAN. Each vertical bar represents a patient (purple progressive and green non-progressive) and each horizontal bar represents a protein (red indicates more abundant proteins while blue less abundant)

In order to analyze whether protein abundances could work as a classifier for progressive vs non-progressive IgAN, we performed receiver operating characteristic (ROC) statistics. First, the top 7 proteins were tested one-by-one; these analyses showed that periostin had the largest area under curve. Thereafter, combinations of proteins were tested, but none of these classifiers significantly improved the area under curve as compared to periostin. Lastly, we combined the 7 top proteins in one classifier and compared this classifier with the one for periostin, cathepsin G and 1/eGFR. As shown in Fig. 4A, AUC values were 0.91 for periostin, 0.91 for the combined top 7 proteins, 0.81 for cathepsin G and 0.72 for 1/eGFR. In order to illustrate protein abundances for these top 7 proteins in the different patients, in Fig. 4B we present a supervised hierarchical clustering map which show moderate separation of progressive and non-progressive IgAN patients.

## Discussion

Little is known about the molecular changes that occur in the tubulointerstitium of patients with IgAN or how these changes may affect progression to kidney failure. To date no studies have investigated whether proteome changes in the tubulointerstitium of IgAN can predict prognosis. In the present study, we microdissected areas with mild tubulointerstitial changes and compared the tubulointerstitial proteome between progressive and non-progressive IgAN patients. The proteins that most significantly predicted a progressive disease course were periostin and cathepsin G. Immunohistochemistry confirmed that periostin staining was stronger in patients with progressive disease, and that periostin was mostly localized in the peritubular areas. Pathway analyses revealed that immune system proteins and rho GTP-ase signaling pathways were upregulated and mitochondrial translation pathways were downregulated in patients with progressive IgAN.

In IgAN nephropathy the immune complexes formed between galactose-deficient IgA1 and antibodies stimulate mesangial cells to secrete various proinflammatory and profibrotic cytokines, components of the extracellular matrix, growth factors, and release reactive oxygen species [12]. These mediators will not only activate neighboring mesangial cells but from other disease models we also know that these could activate proximal tubular epithelial cells (PTECs) [12]. In CKD models in general it is well known that activated PTEC can produce local inflammatory mediators [13] which will attract inflammatory cells, resulting in a positive feedback loop of activation [2]. In our study we show that proteins involved in the immune system are important in progression of IgAN as 23 of the 67 significantly more abundant proteins were related to the immune system. Some of the most abundant proteins involved in the immune system which were significantly more abundant in progressive vs non progressive IgA were cathepsin G, neutrophil gelatinase-associated lipocalin (NGAL) and cathepsin H. Our study did not identify specific pathways within the immune system that seemed to be especially important in IgAN. These findings are in concordance with the findings from our previous studies of glomerular changes in the same group of patients [9, 11]. In these studies, proteins belonging to the immune system also had increase abundance, but different proteins had higher abundance in tubulointerstitium vs glomeruli.

Abundance of tubulointerstitial periostin was nearly nine times higher in progressive vs non-progressive IgAN patients. Periostin is an extracellular protein, lately shown to be involved in both AKI [14] and CKD [15]. In a mice model of unilateral ureteral obstruction, periostin was synthesized by the collecting duct cells and was associated with progression of renal lesions [16]. Increased periostin expression has also been observed in tubular epithelium of patients with diabetic renal disease and urinary periostin levels were also significantly elevated in these patients [17]. In IgAN, higher urinary periostin/creatinine ratios was associated with decreasing eGFR during follow-up and tissue periostin expression was correlated with urinary periostin/creatine ratio and with the renal outcome [18]. Stronger periostin staining by immunohistochemistry in progressive IgAN patients confirmed the proteomic results and localization of the staining was mostly peritubular. In our previous study of glomeruli in IgAN, we also showed that periostin abundance was higher in IgAN as compared to control patients and that higher periostin abundance associated with progressive IgAN [11]. We therefore suggest that periostin should be further investigated as a novel risk marker in IgAN.

In the present study signaling by rho GTP-ases pathway showed increased protein abundances in progressive IgAN as compared with non progressive**.** Rho GTPases contribute to a wide range of cellular processes including organization of the actin and microtubule cytoskeletons, vesicle trafficking, cell cycle progression, cell morphogenesis, cell polarity and cell migration [19]. Rho GTPases have also been shown to be involved in numerous pathologies such as cancer development and progression [20], hypertension [21] and neurodegenerative diseases [22]. In the kidney, Rho-kinase has been shown to be involved in aldosterone-induced renal injury [23], diabetic renal disease [24] and to the pathogenesis of dialysis-related peritoneal fibrosis through epithelial-mesenchymal transition (EMT) [25]. In a model of human renal proximal tubular epithelial cell line, it has been shown that Rho/ROCK signaling pathway plays a key role in the dissolution of tight junctions, an early and reversible event in EMT [26]. EMT is the process by which epithelial cells lose their epithelial proprieties and convert into mesenchymal cells. This mesenchymal cells are then able to migrate, secrete proinflammatory mediators and extracellular matrix (ECM) and thus promote fibrosis. In our study 14 EMT related protein wore more abundant in progressive IgAN as compared with non-progressive.

The mitochondrial translation pathway were also significantly affected in our study. All changed proteins in this pathway were less abundant in progressive IgAN as compared with non-progressive, indicating a mitochondrial dysfunction. Mitochondrial dysfunction is emerging as an important contributor to the development of CKD. The mechanism is not fully understood but it has been suggested that changes in mitochondrial morphology and mitochondrial remodeling, enhanced mitochondrial oxidative stress, and a significant decrease in mitochondrial biogenesis and in ATP production are factors contributing to the progress of CKD [27]. It was unfortunately not possible in our study to investigate this further, for example by further quantification of mitochondrial amount or function.

Our study has several weakness. The main weakness is that number of patients were rather low, but higher numbers were difficult due to the time-consuming and expensive process of microdissection and tandem mass-spectrometry.

We consider it a strength of the study that clinical characteristics did not differ between progressive and non-progressive IgAN patients, but in retrospect we found that three of the patients with progressive IgAN had T-scores of at least one, as compared to none of the non-progressive IgAN patients. It is important to note that patients were selected on the base of similar clinical characteristics, and MEST score was done in retrospect. The significantly different T score underlines the importance of tubular changes in IgAN. In our study we did not microdissected tissue with tubular atrophy or interstitial fibrosis and our findings can thus not be explained by advanced tubulointerstitial changes. We chose to exclude tubuli with atrophy and areas with interstitial expansion as inclusion of such areas would limit the quantitative analysis of protein abundance in our study and also we aimed to investigate factors involved in development of tubulointerstitial damage, and not changes of established tubulointerstitial damage. We therefore believe that our approach is better than a less cautious approach.

In conclusion, our study describes extensive changes of the tubulointerstitial proteome of patients with progressive IgAN. In our opinion, the most interesting finding is that tubulointerstitial periostin seem to be a novel marker of progressive IgAN. This should be investigated further in other cohorts of IgAN and might also be relevant in other kidney diseases.

## Supplementary Information

**Additional file 1:** **Supplemental Table 1.** EMT proteins found in the dbEMT 2.0 database (http://dbemt.bioinfo-minzhao.org/download.cgi). Fold change and *p*-value for difference progressivevs non-progressive IgAN.

## Data Availability

The datasets used and/or analysed during the current study are available from the corresponding author on reasonable request.
