# Supplementary material for: Proteomic signature of tubulointerstitial tissue predicts prognosis in IgAN
Source: BMC Nephrol. 2022 Mar 24;23:118. doi: 10.1186/s12882-022-02736-4 (PMC8943973; doi:10.1186/s12882-022-02736-4)
Supplement: Supplementary file 1 — Additional file 1: Supplemental Table 1. EMT proteins found in the dbEMT 2.0 database (http://dbemt.bioinfo-minzhao.org/download.cgi). Fold change and p-value for difference progressivevs non-progressive IgAN. [file 12882_2022_2736_MOESM1_ESM.docx]

Supplemental Table 1. EMT proteins found in the dbEMT 2.0 database (<http://dbemt.bioinfo-minzhao.org/download.cgi>). Fold change and p-value for difference progressive vs non-progressive IgAN.

| Protein IDs | Gene names | N unique peptides | Protein names | Fold change | p-value |
| --- | --- | --- | --- | --- | --- |
| Q15063 | POSTN | 26 | Periostin | 9.59 | 0.0002 |
| P80188 | LCN2 | 6 | Neutrophil gelatinase-associated lipocalin | 1.67 | 0.004 |
| P63104 | YWHAZ | 17 | 14-3-3 protein zeta/delta | 1.20 | 0.01 |
| P06396 | GSN | 28 | Gelsolin | 1.19 | 0.01 |
| Q9HC35 | EML4 | 18 | Echinoderm microtubule-associated protein-like 4 | 1.15 | 0.02 |
| P60953 | CDC42 | 10 | Cell division control protein 42 homolog | 1.13 | 0.01 |
| Q04760 | GLO1 | 9 | Lactoylglutathione lyase | 1.08 | 0.046 |
| P54646 | PRKAA2 | 4 | 5-AMP-activated protein kinase catalytic subunit alpha-2 | 0.88 | 0.03 |
| P09874 | PARP1 | 12 | Poly ADP-ribose polymerase 1 | 0.68 | 0.03 |
| Q96S96 | PEBP4 | 7 | Phosphatidylethanolamine-binding protein 4 | 1.56 | 0.02 |
| Q9UBX5 | FBLN5 | 7 | Fibulin-5 | 1.22 | 0.03 |
| P22061 | PCMT1 | 7 | Protein-L-isoaspartate(D-aspartate) O-methyltransferase | 1.11 | 0.04 |
| P35754 | GLRX | 11 | Glutaredoxin-1 | 0.91 | 0.047 |
| P30086 | PEBP1 | 23 | Phosphatidylethanolamine-binding protein 1 | 1.30 | 0.048 |
